# New colleague or gimmick hurdle? A user-centric scoping review of the barriers and facilitators of robots in hospitals

**DOI:** 10.1371/journal.pdig.0000660

**Published:** 2024-11-11

**Authors:** Mathias Kofoed Rasmussen, Anna Schneider-Kamp, Tobias Hyrup, Alessandro Godono

**Affiliations:** 1 Department of Business & Management, University of Southern Denmark, Denmark; 2 Department of Mathematics and Computer Science, University of Southern Denmark, Denmark; 3 Department of Public Health and Pediatrics, University of Torino, Italy; The London School of Economics and Political Science, UNITED KINGDOM OF GREAT BRITAIN AND NORTHERN IRELAND

## Abstract

Healthcare systems are confronted with a multitude of challenges, including the imperative to enhance accessibility, efficiency, cost-effectiveness, and the quality of healthcare delivery. These challenges are exacerbated by current healthcare personnel shortages, prospects of future shortfalls, insufficient recruitment efforts, increasing prevalence of chronic diseases, global viral concerns, and ageing populations. To address this escalating demand for healthcare services, healthcare systems are increasingly adopting robotic technology and artificial intelligence (AI), which promise to optimise costs, improve working conditions, and increase the quality of care. This article focuses on deepening our understanding of the barriers and facilitators associated with integrating robotic technologies in hospital environments. To this end, we conducted a scoping literature review to consolidate emerging themes pertaining to the experiences, viewpoints perspectives, and behaviours of hospital employees as professional users of robots in hospitals. Through screening 501 original research articles from Web-of-Science, we identified and reviewed in full-text 40 pertinent user-centric studies of the integration of robots into hospitals. Our review revealed and analysed 14 themes in-depth, of which we identified seven as barriers and seven as facilitators. Through a structuring of the barriers and facilitators, we reveal a notable misalignment between these barriers and facilitators: Finding that organisational aspects are at the core of most barriers, we suggest that future research should investigate the dynamics between hospital employees as professional users and the procedures and workflows of the hospitals as institutions, as well as the ambivalent role of anthropomorphisation of hospital robots, and emerging issues of privacy and confidentiality raised by increasingly communicative robots. Ultimately, this perspective on the integration of robots in hospitals transcends debates on the capabilities and limits of the robotic technology itself, shedding light on the complexity of integrating new technologies into hospital environments and contributing to an understanding of possible futures in healthcare innovation.

## Author summary

Healthcare systems are confronted with a multitude of challenges, including the imperative to enhance accessibility, efficiency, cost-effectiveness, and the quality of healthcare delivery. These challenges are exacerbated by current healthcare personnel shortages, prospects of future shortfalls, insufficient recruitment efforts, increasing prevalence of chronic diseases, global viral concerns, and ageing populations. To address this escalating demand for healthcare services, healthcare systems are increasingly adopting robotic technology and artificial intelligence (AI), which promise to optimise costs, improve working conditions, and increase the quality of care. This article identifies seven barriers and seven facilitators associated with integrating robotic technologies in hospital environments through a scoping review of the academic literature. As most barriers are rooted in organisational aspects, we suggest that researchers should investigate the dynamics between hospital employees and the procedures and workflows of the hospitals. They should also look into aspects such as users humanizing hospital robots and emerging issues of privacy and confidentiality raised by increasingly communicative robots to understand how robots can positively contribute to improving future healthcare systems.

## 1. Introduction

On a global scale, healthcare systems are being challenged to increase accessibility and efficiency, optimise cost structures, and maintain and further develop the quality of healthcare delivery [1]. These challenges are aggravated in light of a number of exacerbating factors. There is a current shortage of healthcare personnel, prospects of an even more pronounced shortage of healthcare personnel in the future, and a demonstrated insufficiency of existing recruitment efforts [2]. Furthermore, the growth in chronic disease prevalence and global attention towards viruses [1,3,4] and the prospects of rapidly ageing populations [5] can be assumed to further increase the demand for healthcare services and the ensuing pressure on healthcare systems.

Addressing this demand, healthcare systems have been introduced to the application of robotic technology and artificial intelligence (AI) in a multitude of healthcare environments. The implementation of these rapidly advancing technologies in healthcare environments is often associated with expectations of optimisation within the areas of cost efficiency, working environment, and patient care [6]. During the last decades, both the academic and practitioner sectors have witnessed growing attention to and application of robots within healthcare. At the beginning of the century, the da Vinci Surgical System, a robotic-assisted platform for minimally invasive surgery where surgeons control instruments through a console [7], gained FDA approval, which paved the way for numerous innovative applications of robotic technology and AI [8]. Since then, robots have been deployed in a wide variety of healthcare environments, including, but not limited to, hospitals, elderly care, and rehabilitation centres [9].

On one hand, researchers and developers have widely advocated the present and potential future benefits of the application of robots in healthcare [10–12]. The prospects of increasing the prevalence of robots within healthcare have been touted as a key facilitator towards managing the current and future ‘care crisis’ [6]. On the other hand, research and practical evaluations have highlighted a number of significant challenges hindering the user experience and perceived utility of robots in healthcare environments [13,14], thereby hindering the realisation of promises and expectations of progress in the area [15]. Considering the increasing use of AI as an integral part of robotic systems, the perceived utility can also be assumed to suffer from biases in training data such as representation and collection bias. Such biases are hard to assess for end users, further exacerbating their potential impact. Approaches for mitigating bias in training data represent an active research area [16].

### 1.1. Aim of the study

The investigation of robotic technology and AI integration within healthcare has become a prominent research topic, as academics have started to examine different stakeholders, distinct types of robots and AI solutions, and barriers and facilitators of successful integration [9,11,17]. However, these studies have predominantly paid attention to the environment of elderly care [18,19] and the perspectives of patients and citizens as end-users [6,20]. While the literature increasingly reports on research in hospital environments and the perspective of hospital employees as professional users of robots, this budding evidence has not yet been extensively reviewed and structured.

Potential benefits of integrating robots within hospitals to optimise and streamline healthcare delivery are reported alongside contradictory viewpoints regarding the unpredictable challenges experienced by professional users and the ensuing lack of perceived utility [8,12]. The complexity of multi-stakeholder scenarios in hospital environments, the accompanying cost of investments, and the neglect of the professional end-users’ concerns represent major causes of failure in the integration of robots in hospital contexts [1,21].

In this article, we aim to *enhance our understanding of the barriers to and facilitators of the integration of robots in hospital environments* and to map and summarise the evidence on this topic. To this end, we performed a scoping literature review, collecting and integrating evidence-based emerging themes regarding the experiences, viewpoints, perspectives, and behaviours of professional users of robots in hospitals.

### 1.2. Types of robots

Extant research on robots in hospitals has investigated the utility and characteristics associated with a broad spectrum of robots. We briefly summarise the three main types of robots encountered in hospital environments.

*Social robots* have been designed, programmed, and deployed to provide social qualities and attributes such as interaction, engagement, and companionship. They often incorporate components that mimic human behaviour and simulate social interactions through gestures, speech, and/or anthropomorphic movements [4,14] and target cognitively impaired, elderly citizens, and/or children for positive therapeutic impacts on well-being and cognitive training [14,22]. Pet robots imitate animals’ appearances and behaviour and provide psychosocial and emotional benefits for persons living with dementia [15] or children undergoing medical treatment [23]. Telepresence robots comprise a screen and speakers and act as a mobile video conferencing platform [4], facilitating social interactions between patients, hospital employees, and relatives, as well as remote-assisted treatment of trauma and general surgery [4,24].

*Service robots* have been designed to assist in the streamlining of supportive service activities such as janitorial or logistical tasks such as cleaning or the delivery of medicine, food, blood samples, and bed linen [1,25]. Such robots have the potential to increase the productivity of hospital employees [1]. The interest in service robots is growing with increasing capabilities and decreasing costs, as such robots offload logistic activities, reduce operational costs, and improve the efficiency of everyday processes [26].

Another type of robot are *clinical robots* that are applied to maintain high levels of hygiene and sterility [6,27,28] or to directly assist hospital employees in their treatment and diagnostics of patients [7,29]. Rehabilitation robots have received recognition for their ability to aid patients in overcoming physical impairments [9]. Such clinical robots assist patients in the performance of repetitive tasks and the collection of data through the robot’s sensors, allowing for detailed analyses of treatment progress and efficacy [30,31].

## 2. Methods

This scoping literature review was conducted through the guidance and instructions proposed by the Preferred Reporting Items for Systematic Review and Meta-Analysis extension for Scoping Reviews (PRISMA-ScR) [32] and the JBI Manual for Evidence Synthesis [33], to ensure a transparent, explicit, and comprehensive level of reporting. The PRISMA-ScR checklist is available in the **S1 Appendix**. The review aimed at a qualitative synthesis, did not involve the collection of personal data, did not apply statistical methods such as meta regression, and was exempt from any registration and ethical approval requirements. None of the authors have any conflicts of interest.

### 2.1. Eligibility criteria

A set of five predetermined eligibility criteria were applied to determine the studies’ relevancy and validity:

1. To ensure the continued relevance of the findings in a fast-moving technological field, the studies must be published after 1 January 2009.
2. The studies must be accessible and written in English.
3. The studies must be peer-reviewed.
4. The studies must clearly reference or investigate hospital employees.
5. The studies must present or reference an exploration of end-users’ experiences, perspectives, and behaviours regarding the application of robots in care or nursing practices.

### 2.2. Search strategy

An initial literature search was conducted in March 2023, with the intent of establishing insights into the balance between sensitivity and specificity [34], i.e., between developing a comprehensive search string able to identify a sufficient number of studies and restrictions to search terms that yield a large percentage of relevant studies. This initial search facilitated a better understanding of the commonly used terminology within the given research subject and aided in the development of a more optimal search strategy. The initial search further offered insights into how the main subjects, robotics and hospitals, cross several research areas. Each of the two subjects on its own represents an established and rich research area, comprised of an extensive amount of academic literature with empirical studies numbering in the thousands. In light of this volume of potential search results, for the final search, we refrained from searching multiple databases [34] and followed best practices to rely on the Web-of-Science (WoS) database with its focus on high-quality journal articles as the most appropriate source ‘to curate a manageable collection of articles for review when researchers encounter thousands of articles’ [35]. Taking the qualitative nature of our review into account, this final search yielded clearly sufficient numbers of articles for full-text review and inclusion, particularly when benchmarking against comparable qualitative reviews [36]. As common in qualitative research, from a certain point in our analysis, we observed theoretical saturation [37], i.e., further articles supported rather than extended the themes emerging from our qualitative synthesis.

The final literature search was conducted in May 2023, by searching WoS’ Core Collection; thus, ensuring that all the identified studies were peer-reviewed, which subsequently fulfilled eligibility Criterion 3. Through the literature search, we identified 501 studies after the exclusion of one duplicate. The precise search string used was the following:

ALL = ("attitude*" OR "perspective*" OR "point of view" OR "nurse*" OR "healthcare worker" OR "healthcare-professional" OR "health worker" OR "user experience" OR "barrier*" OR "challenge*" OR "user perspectives" OR "user*" OR "facilitators" OR "implementation" OR "enabler*" OR "healthcare" OR "influenc*" OR "key factors" OR "impact*" OR "benefits" OR "problems" OR "perception*" OR "adapt*" OR "compliance" OR "behavior*" OR "accept*" OR "assistive technology" OR "opinion*") AND ALL = ("robots" AND "hospital")

The disjunctive and conjunctive terms of the search string are illustrated in the **S1 Table**.

### 2.3. Screening

The identified studies underwent a selection process consisting of three screening stages conducted by at least two authors with the help of the collaborative review platform Covidence [38]. The intent of each stage was to exclude studies deemed irrelevant through screening distinctive parts of the studies, for each study assessing whether it complies with the eligibility criteria. First, we screened the studies’ titles and excluded studies with titles clearly deemed irrelevant. Second, we screened the remaining studies’ abstracts and excluded the clearly irrelevant ones. Third and last, we screened the remaining studies’ full texts and retained only the studies that we deemed relevant and that comply with all the eligibility criteria for subsequent synthesis. Backwards snowballing was applied in instances of review papers which could not fulfil Criterion 5 to an adequate extent by including the referenced studies. After title, abstract, and full-text screening, as well as backward snowballing, of the initial 501 identified studies, a total of 40 complied with the eligibility criteria.

**Fig 1:**
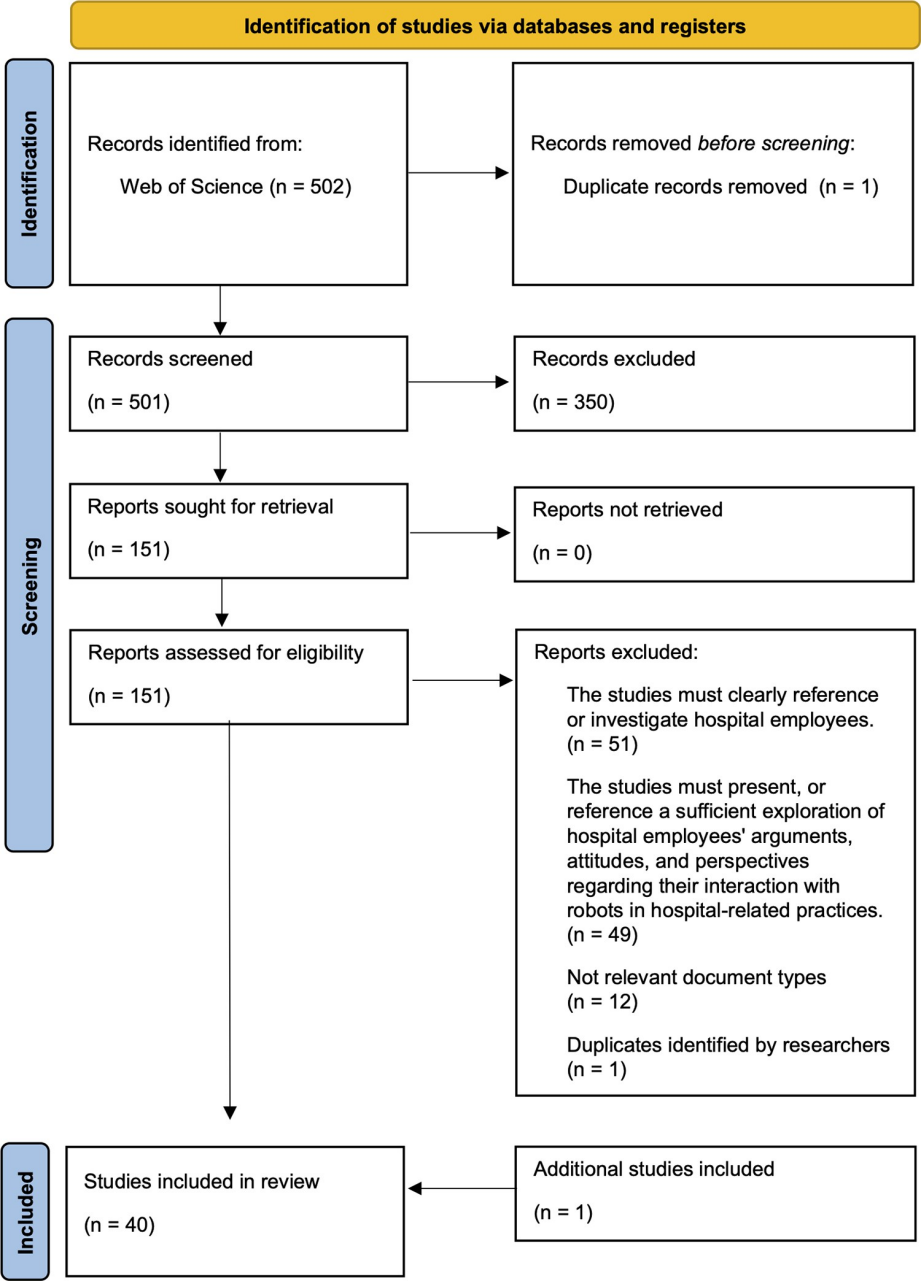
PRISMA chart visualising the article selection process.

The screening process is summarised and illustrated in **Fig 1** through a PRISMA chart. The main bibliographical details and study designs of the 40 included studies are summarised in **Table 1**, with consecutive reference numbers to identify particular articles in later tables.

**Table 1:** Overview of bibliometric details and study design of the 40 included studies. We marked high-income countries according to the World Bank’s income groups with one asterisk (‘*’) and upper-middle-income countries with two asterisks (‘**’).

Ref.	Title of study	Authors and year	Publ. type	Journal/Conference title	Study design	Prof. users	Country
S1	A Long-Term Autonomous Robot at a Care Hospital: A Mixed Methods Study on Social Acceptance and Experiences of Staff and Older Adults	Hebesberger et al., 2017	Journal paper	International Journal of Social Robotics	Interview + Questionnaire	Administrative staff, Physical therapists, Physicians	Austria*
S2	A Robot-Mediated Activity Using the Nao Robot to Promote COVID-19 Precautionary Measures among Older Adults in Geriatric Facilities	Blavette et al., 2022	Journal paper	International Journal of Environmental Research and Public Health	Questionnaire	Healthcare professionals	France*
S3	A Survey of Nurses’ Need for Care Robots in Children’s Hospitals: Combining Robot-Care, Game-Care, and Edu-Care	Jin & Kim, 2020	Journal paper	CIN: Computers, Informatics, Nursing	Questionnaire + Interviews	Nurses	South Korea*
S4	A Survey of Robots in Healthcare	Kyrarini et al., 2021	Journal paper	Technologies	Survey paper	Nursing staff, Physical therapists	N/A
S5	Acceptability of Using a Robotic Nursing Assistant in Health Care Environments: Experimental Pilot Study	Saadatzi et al., 2020	Journal paper	Journal of Medical Internet Research	Questionnaire	Nursing students	United States*
S6	ARI: the Social Assistive Robot and Companion	Cooper et al., 2020	Conference paper	29^th^ IEEE International Conference on Robot and Human Interactive Communication	Review	Nursing staff, Physicians	N/A
S7	Artificial intelligence and robot nurses: From nurse managers’ perspective: A descriptive cross-sectional study	Ergin et al., 2022	Journal paper	Journal of nursing management	Questionnaire	Nursing staff	Turkey**
S8	Artificial Intelligence in Healthcare Robots: A Social Informatics Study of Knowledge Embodiment	Pee et al., 2019	Journal paper	Journal of the Association for Information Science and Technology	Interviews	Nurses, Physicians, Administrative staff	China**
S9	Barriers and facilitators in the implementation of mobilization robots in hospitals from the perspective of clinical experts and developers	Warmbein et al., 2023	Journal paper	BMC Nursing	Interviews	Nurses, Physical therapists	Austria*, Denmark*, Germany*, Switzerland*
S10	Determining the feasibility of robotic courier medication delivery in a hospital setting	Kirschling et al., 2009	Journal paper	American journal of health-system pharmacy	Pilot study	Nursing staff, Pharmaceutical staff, Administrative staff	United States*
S11	Development and usability evaluation of a bedside robot system for inpatients	Yoo et al., 2022	Journal paper	Technology and Health Care	Survey + interviews	Nurses	South Korea*
S12	Digital skills in context: Working with robots in lower-skilled jobs	Lloyd & Payne, 2022	Journal paper	Economic and industrial democracy	Interviews	Service staff	Norway*, United Kingdom*
S13	Embedding robotic surgery into routine practice and impacts on communication and decision making: a review of the experience of surgical teams	Randell et al., 2016	Journal paper	Cognition, Technology & Work	Review	N/A	N/A
S14	Expectations and Perceptions of Healthcare Professionals for Robot Deployment in Hospital Environments During the COVID-19 Pandemic	Sierra Marin et al., 2021	Journal paper	Frontiers in robotics and AI	Survey	Nurses, Physical therapists, Physicians, Technical staff	Colombia**
S15	Factors influencing acceptance of robotics in hospital pharmacy: a longitudinal study using the Extended Technology Acceptance Model	Hogan et al., 2020	Journal paper	International Journal of Pharmacy Practice	Survey	Pharmaceutical staff	Australia*
S16	Health Promotion, Health Literacy and Vaccine Hesitancy: The Role of Humanoid Robots	McIntosh et al., 2022	Journal paper	The Journal of Health Care Organization, Provision, and Financing	Interviews	Nurses, Physicians	Australia*
S17	Healthcare Workers’ Point of View on Medical Robotics During COVID-19 Pandemic–A Scoping Review	Defi et al., 2022	Journal paper	International Journal of General Medicine	Scoping review	Nurses, Physicians	N/A
S18	How Robots Help Nurses Focus on Professional Task Engagement and Reduce Nurses’ Turnover Intention	Chang et al., 2021	Journal paper	Journal of Nursing Scholarship	Survey	Nurses	Taiwan*
S19	How robots impact nurses’ time pressure and turnover intention: A two-wave study	Huang et al., 2022	Journal paper	Journal of nursing management	Observations	Nurses	Taiwan*
S20	Implementation of robotic devices in nursing care. Barriers and facilitators: an integrative review	Servaty et al., 2020	Journal paper	BMJ open	Integrative review	N/A	N/A
S21	Investigating human-robot cooperation in a hospital environment	Tornbjerg et al., 2021	Conference paper	Proceedings of the 2021 Acm Designing Interactive Systems Conference	Interviews + Observations	Healthcare professionals, Kitchen staff, Service staff	Denmark
S22	Nurses’ Views on the Potential Use of Robots in the Pediatric Unit	Liang et al., 2020	Journal paper	Journal of Pediatric Nursing	Interviews	Nurses	Taiwan*
S23	Reconfiguring Boundary Relations: Robotic Innovations in Pharmacy Work	Barrett et al., 2012	Journal paper	Organization Science	Observation + Interview	Pharmaceutical staff	United Kingdom*
S24	Reducing Loneliness in Stationary Geriatric Care with Robots and Virtual Encounters—A Contribution to the COVID-19 Pandemic	Follmann et al., 2021	Journal paper	International journal of environmental research and public health	Observation + Questionnaire	Nurses	Germany*
S25	Robotic Assistance in Coordination of Patient Care	Gombolay et al., 2018	Journal paper	The International Journal of Robotics Research	Questionnaire	Nurses	United States*
S26	Robotic Systems in Operating Theaters: New Forms of Team–Machine Interaction in Health Care On Challenges for Health Information Systems (…)	Steil et al., 2019	Journal paper	Methods of Information in Medicine	Position paper	N/A	N/A
S27	Scenario-Based Assessment of User Needs for Point-of-Care Robots	Lee & Kim, 2018	Journal paper	Healthcare Informatics Research	Survey + Interviews + Observations	Nurses, Physicians	South Korea*
S28	Service robots in hospitals: new perspectives on niche evolution and technology affordances	Mettler et al., 2017	Journal paper	European Journal of Information Systems	Observations + Interviews	Nurses, Physician	Switzerland*
S29	Service Robots in the Healthcare Sector	Holland et al., 2021	Journal paper	Robotics	Review	N/A	N/A
S30	Social domestication of service robots: The secret lives of Automated Guided Vehicles (AGVs) at a Norwegian hospital	Soraa & Fostervold, 2021	Journal paper	International Journal of Human-Computer Studies	Interviews + observations	Cleaning staff, Nursing staff, Physicians	Norway*
S31	Teaching Hospitals and the Disconnect Between Technology Adoption and Comparative Effectiveness Research: The Case of the Surgical Robot	Makarov et al., 2016	Journal paper	Medical care research and review	Review	N/A	N/A
S32	The Increasing Centrality of Robotic Technology in the Context of Nursing Care: Bioethical Implications Analyzed through a Scoping Review Approach	Gibelli et al., 2021	Journal paper	Journal of Healthcare Engineering	Scoping review	N/A	N/A
S33	The requirements and applications of autonomous mobile robotics (AMR) in hospitals from the perspective of nursing officers	Kriegel et al., 2022	Journal paper	International Journal of Healthcare Management	Interviews + Survey	Nurses	Austria*
S34	The Role of Humans in Surgery Automation	Fosch-Villaronga et al., 2023	Journal paper	International Journal of Social Robotics	Review	N/A	N/A
S35	The Use of Affective Care Robots Calls Forth Value-based Consideration	Turja & Parviainen, 2020	Conference paper	29^th^ IEEE International Conference on Robot and Human Interactive Communication	Survey	Nurses	Finland*
S36	The use of robotics in surgery: a review	Hussain et al., 2014	Journal paper	The International Journal of Clinical Practise	Review	N/A	N/A
S37	Usability Evaluation of User Requirement–Based Teleconsultation Robots: A Preliminary Report from South Korea	Lee et al., 2020	Journal paper	Methods of Information in Medicin	Survey + Interviews	Nurses, Physicians	South Korea*
S38	Use of pharmacy delivery robots in intensive care units	Summerfield et al., 2011	Journal paper	American journal of health-system pharmacy	Interviews + Survey + Observations	Nurses	United States*
S39	User-centered Exploration of Robot Design for Hospitals in COVID-19 Pandemic	Shin et al., 2022	Conference paper	Proceedings of the 17^th^ ACM/IEEE International Conference on Human-Robot Interaction	Interviews	Nurses	South Korea*
S40	User Validation Study of a Social Robot for Use in Hospital Wards	Ramachandran & Lim, 2021	Conference paper	ACM/IEEE International Conference on Human-Robot Interaction	Survey	Nurses	Singapore*

### 2.4. Quality assessment

The eligible studies were assessed by applying the GRADE-CERQual approach (Grading of Recommendations Assessment, Development and Evaluation—Confidence in Evidence from Reviews of Qualitative Research), to ensure a transparent assessment of the studies’ quality [39]. The purpose of applying the GRADE-CERQual approach to a study is to address to what degree potential readers, users, or decision-makers, may assume the findings to be representative of the phenomenon studied. This approach offers a framework that ensures the researcher a rigorous and transparent process of assessing confidence in individual review findings [39]. The framework is based on evaluating and considering four components: methodological limitations, coherence, adequacy of data, and relevance. Each component should be evaluated by applying a four-degree scale consisting of the following levels: no or very minor concerns, minor concerns, moderate concerns, and serious concerns [40]. By assessing these components collectively, an overall assessment of the findings can be constructed, which determines whether they contribute to a reasonable and cogent representation of the research subject.

### 2.5. Analysis

The full text of each eligible study was examined following an inductive thematic synthesis methodology inspired by extant reviews of qualitative studies [36]. We aimed to develop analytical themes of the hospital employees’ arguments in relation to utilising and integrating robots. We performed the thematic synthesis in three phases: (a) line-by-line coding, (b) developing descriptive themes, and (c) generating analytical themes.

## 3. Results

Half of the included studies followed a qualitative methodological approach through either interviews, observations, or surveys (20/40). A few studies each applied a quantitative (4/40) and a mixed-methods (4/40) approach. The remaining studies were predominantly different types of reviews (9/40), position and survey papers (2/40), and a pilot study (1/40).

**Fig 2:**
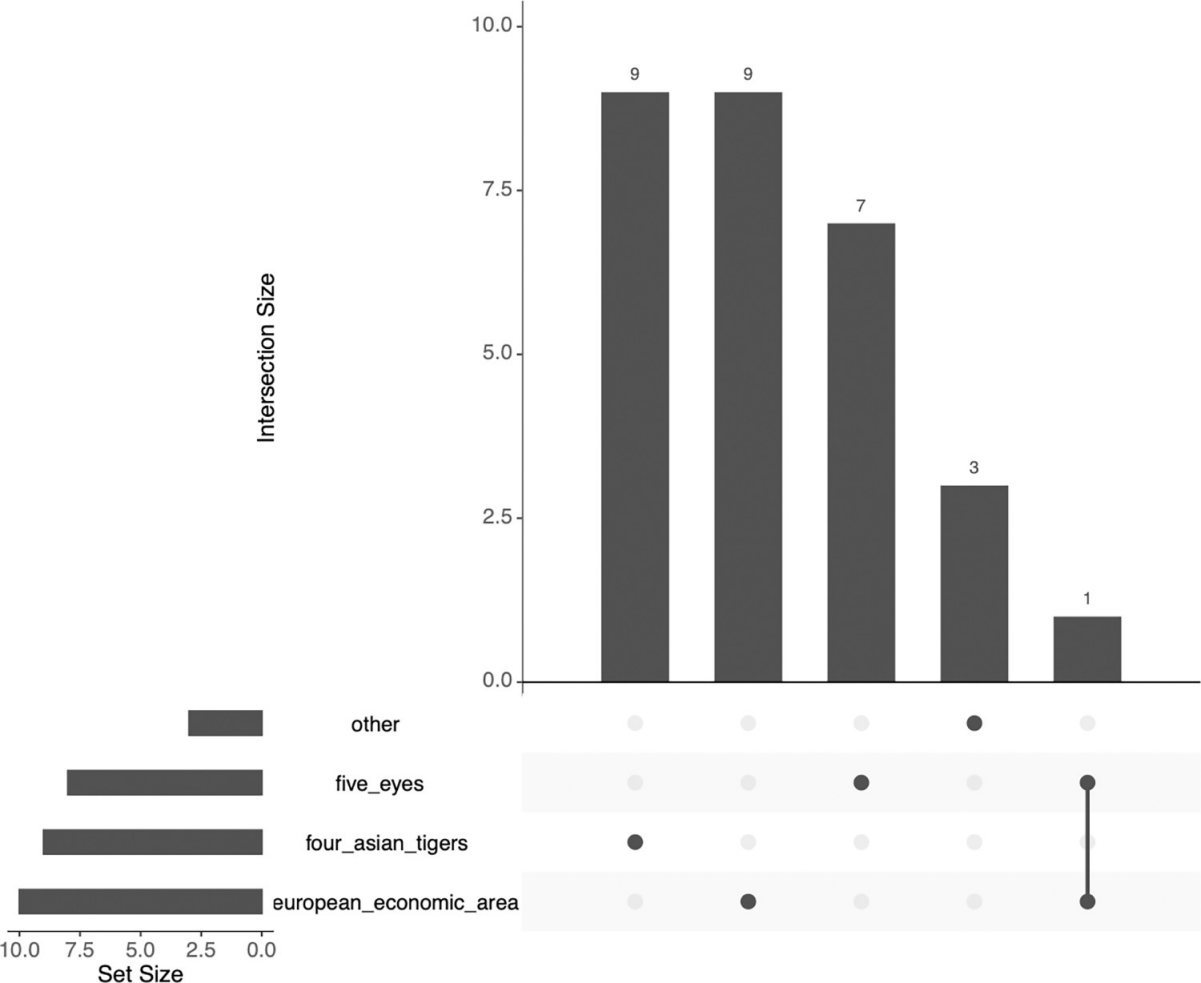
Country contexts of the included studies.

As indicated in **Table 1**, the countries of the studies were predominantly high-income countries, except for three upper-middle-income countries. All these countries exhibit high societal and technological readiness levels regarding integrating robots in hospital environments. The distribution of studies by country context is visualised in **Fig 2**, where the ‘Four Asian Tigers’ [41] include Singapore, South Korea, and Taiwan while the ‘Five Eyes’ [42] include Australia, the United Kingdom, and the United States. The remaining countries are either high-income countries from the ‘European Economic Area’ or the upper-middle-income countries China, Colombia, and Turkey grouped as ‘Other’.

**Fig 3:**
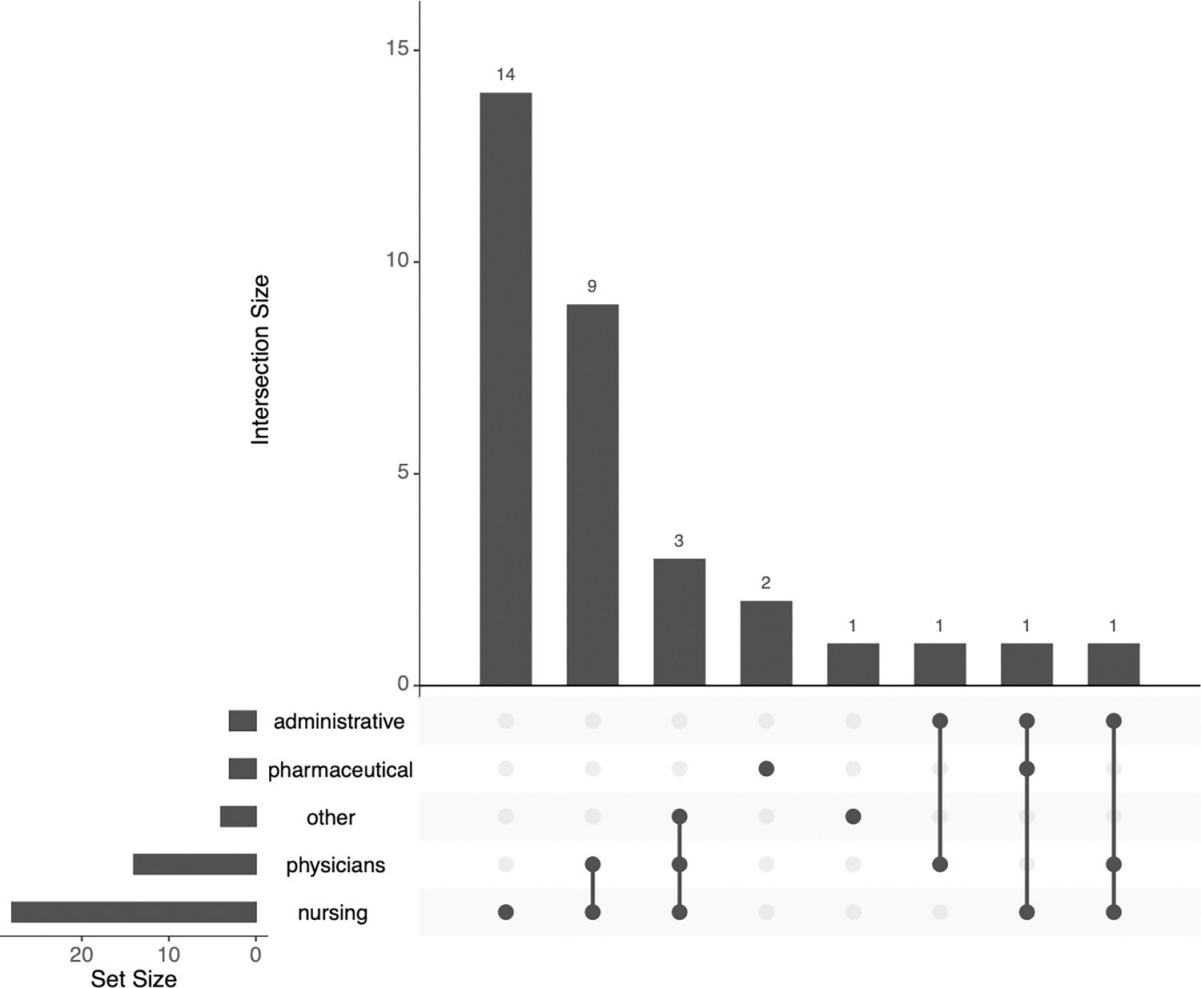
Professional roles of end-users of the included studies.

Furthermore, in **Table 1**, we also indicate which type of professional end-users the included studies focused on. To visualise the results, in **Fig 3** we group the professional roles into five categories, where ‘nursing’ covers nursing students, nurses, and nursing staff while ‘other’ covers cleaning, kitchen, service, and technical staff and ‘physicians’ also includes physical therapists. Most of the included studies focus on nurses (alone or in combination with physicians).

**Table 2** illustrates the Grade-CERQual summary of qualitative findings (SoQF) table, presenting the barriers and facilitators of applying and integrating robots within a hospital environment, developed from the thematic synthesis of the included studies. The table offers an evaluation of the degree of confidence towards the identified evidence relating to the barriers, facilitators, and the findings associated with them.

**Table 2:** Barriers and facilitators of integrating robots within a hospital domain. The first column summarises the evidence of the studies indicated in the second column. The third and fourth columns provide the GRADE-CERQual assessment of the confidence in this evidence.

Analytical themes and findings	Contributing studies (Reference numbers refer to Table 1)	Confidence in evidence	Explanation of confidence in evidence assessment
**BARRIERS**			
**B1: USER PERCEPTIONS**	**11 studies**		
End-users were impacted by attitudes of resentment and scepticism towards robots.	S1, S7, S9, S10, S15, S21, S22, S29, S32, S33, S35	High	1 study with minor and 2 studies with moderate concerns regarding methodological limitations. 4 studies with minor and 3 studies with moderate concerns regarding coherence. 5 studies with minor and 1 study with moderate concerns regarding adequacy. 4 studies with minor and 1 study with moderate concerns regarding relevance.
End-users were afraid of being replaced.	S1, S7, S9, S22, S29	High	1 study with moderate concerns regarding methodological limitations. 1 study with minor and 1 study with moderate concerns regarding coherence. 1 study with minor and 1 study with moderate concerns regarding adequacy. 1 study with moderate concerns regarding relevance.
End-users associated robots with a decrease in their own competences and skill sets.	S7, S22	Moderate	No concerns.
End-users rejected any use of robots.	S1, S9, S35	Moderate	1 study with moderate concerns regarding methodological limitations. 1 study with minor and 1 study with moderate concerns regarding coherence. 2 studies with minor concerns regarding adequacy. 1 study with minor concerns regarding relevance.
**B2: INSUFFICIENT KNOWLEDGE, AWARENESS, AND SUPPORT**	**14 studies**		
End-users were concerned by the lack of resources, information, and assistance.	S1, S6, S7, S8, S9, S12, S14, S21, S23, S26, S28, S31, S32, S34	High	1 study with minor and 2 studies with moderate concerns regarding methodological limitations. 1 study with minor and 3 studies with moderate concerns regarding coherence. 1 study with minor and 2 studies with moderate concerns regarding adequacy. 5 studies with minor and 1 study with moderate concerns regarding relevance.
End-users had limited learning opportunities and poor understandings of functions, features, and utility purposes.	S7, S9, S14, S23, S26, S32, S34	High	1 study with minor concerns regarding methodological limitations. 2 studies with minor and 1 study with moderate concerns regarding coherence. 2 studies with minor and 1 study with moderate concerns regarding adequacy. 3 studies with minor and 1 study with moderate concerns regarding relevance.
The lack of knowledge contributed to feeling nervous, underprepared, and doubtful of the robot’s utilitarian value.	S9, S23, S32	Medium	No concerns regarding methodological limitations. 1 study with minor and 1 study with moderate concerns regarding coherence. 2 studies with minor concerns regarding adequacy. 1 study with minor concerns regarding relevance.
End-users had little awareness of the robot due to missing initiative and proper introduction.	S8, S14, S21, S23	High	No concerns regarding methodological limitations and coherence. 2 studies with minor concerns regarding adequacy. 1 study with minor concerns regarding relevance.
**B3: INADEQUATE IMPACT ON HEALTHCARE PRACTICES**	**10 studies**		
End-users were concerned and dissatisfied with the robot’s limited usefulness.	S1, S5, S9, S10, S11, S21, S22, S23, S28, S38	High	3 studies with minor and 1 study with moderate concerns regarding methodological limitations. 4 studies with minor and 1 study with moderate concerns regarding coherence. 4 studies with minor and 1 study with moderate concerns regarding adequacy. 3 studies with minor concerns regarding relevance.
The robot led to an increase in workload and frequency of errors.	S10, S11, S22, S23, S38	High	2 studies with minor and 1 study with moderate concerns regarding methodological limitations. 2 studies with minor and 1 study with moderate concerns regarding coherence. 2 studies with minor and 1 study with moderate concerns regarding adequacy. 3 studies with minor concerns regarding relevancy.
End-users perceived the robots as unreliable and were uncomfortable using them.	S21, S38	Low	1 study with minor concerns regarding methodological limitations, coherence, adequacy, and relevance.
**B4: LIMITATIONS TO FUNCTIONALITY AND DESIGN**	**10 studies**		
End-users were concerned with the robot’s design features and subpar functionalities.	S1, S2, S9, S10, S20, S22, S28, S30, S32, S40	High	3 studies with minor and 1 study with moderate concerns regarding methodological limitations. 5 studies with minor and 1 study with moderate concerns regarding coherence. 6 studies with minor and 1 study with moderate concerns regarding adequacy. 5 studies with minor concerns regarding relevance.
End-users were frustrated with outages and malfunctions.	S 1, S9, S20	Moderate	1 study with minor concerns regarding methodological limitations. 2 studies with minor concerns regarding coherence. 2 studies with minor concerns regarding adequacy. 1 study with minor concerns regarding relevance.
Robots with verbal communicative features could lead to problems of confidentiality and privacy violations.	S2, S22, S32	Moderate	1 study with minor concerns regarding methodological limitations. 1 study with minor and 1 study with moderate concerns regarding coherence. 2 studies with minor concerns regarding adequacy. 2 studies with minor concerns regarding relevance.
Challenging adaptation to complex facilities and infrastructure.	S9, S20, S28, S30	High	1 study with minor concerns regarding methodological limitations. 2 studies with minor concerns regarding coherence. 3 studies with minor concerns regarding adequacy. 1 study with minor concerns regarding relevance.
**B5: WORKPLACE DYNAMICS**	**6 studies**		
Robots complicated the distribution of responsibility and roles.	S7, S17, S21, S23, S34, S38	High	1 study with minor concerns regarding methodological limitations. 2 studies with minor concerns regarding coherence. 2 studies with minor concerns regarding adequacy. 2 studies with minor concerns regarding relevance.
The end-users involuntary had to accommodate to new roles and tasks.	S21, S23, S38	Moderate	1 study with minor concerns regarding methodological limitations, coherence, adequacy, and relevance.
**B6: WORKFLOW IMPLEMENTATION**	**7 studies**		
End-users experienced difficulties when adopting robots into routines and work processes.	S9, S10, S13, S21, S23, S28, S30	High	1 study with minor concerns regarding methodological limitations. 2 studies with minor concerns regarding coherence. 3 studies with minor concerns regarding adequacy. 1 study with minor concerns regarding relevance.
The adaptation process of robots led to disruptions and exacerbation in quality of care.	S9, S13, S21, S30	High	No concerns regarding methodological limitations. 1 study with concerns regarding coherence. 2 studies with minor concerns regarding adequacy. High relevance.
**B7: LOSS OF CONTROL AND AUTHORITY**	**4 studies**		
The end-users perceived the robot as unpredictable and felt an unpleasant notion of dependency towards them.	S1, S23, S30, S32	Moderate	No concerns regarding methodological limitations. 1 study with moderate concerns regarding coherence. 2 studies with minor concerns regarding adequacy. 1 study with minor concerns regarding relevance.
**FACILITATORS**			
**F1: TRAINING AND ENGAGEMENT**	**12 studies**		
Extensive training and engagement activities improved the robots’ perceived utility and overall satisfaction levels.	S7, S9, S12, S13, S15, S20, S27, S29, S31, S32, S34, S40	High	3 studies with minor and 2 studies with moderate concerns regarding methodological limitations. 3 studies with minor and 5 studies with moderate concerns regarding coherence. 6 studies with minor and 2 studies with moderate concerns regarding adequacy. 3 studies with minor and 4 studies with moderate concerns regarding relevancy.
The end-users appreciated a comprehensive understanding of the robot’s features and complexity.	S9, S12, S20, S29, S32, S34	High	1 study with minor and 1 study with moderate concerns regarding methodological limitations. 2 studies with minor and 2 studies with moderate concerns regarding coherence. 4 studies with minor and 1 study with moderate concerns regarding adequacy. 3 studies with minor and 1 study with moderate concerns regarding relevancy.
Public campaigns, consultation, implementation strategies, and involvement of end-users influenced the robot’s perception, intention, and usefulness.	S9, S13, S15, S20, S27, S29, S32,	Moderate	2 studies with minor and 1 study with moderate concerns regarding methodological limitations. 3 studies with minor and 3 studies with moderate concerns regarding coherence. 5 studies with minor and 1 study with moderate concerns regarding adequacy. 2 studies with minor and 2 studies with moderate concerns regarding relevancy.
**F2: TRANSPARENCY**	**10 studies**		
End-users were appreciative of an insight into the robot’s utility, roles, responsibilities, and expectations.	S7, S9, S13, S15, S20, S23, S30, S32, S33, S34	High	1 study with minor concerns regarding methodological limitations. 4 studies with minor and 1 study with moderate concerns regarding coherence. 6 studies with minor concerns regarding adequacy. 3 studies with minor concerns regarding relevance.
End-users appreciated clarity regarding the robot’s limitations and responsibilities, and the potential impact on work processes and roles.	S7, S9, S13, S15, S20, S32, S33, S34	High	1 study with minor concerns regarding methodological limitations. 3 studies with minor and 1 study with moderate concerns regarding coherence. 4 studies with minor concerns regarding adequacy. 3 studies with minor concerns regarding relevance.
Presenting robots as an assisting tool led to a decrease in doubts and uncertainties regarding fear of being replaced.	S9, S30, S34	Moderate	No concerns regarding methodological limitations. 1 study with minor concerns regarding coherence. 2 studies with minor concerns regarding adequacy. High relevancy.
**F3: BEHAVIOURAL AND CULTURAL FACTORS**	**10 studies**		
Favorable attitudes, perceptions, and values among end-users, had an enabling impact on the application and integration of robots.	S1, S9, S11, S13, S15, S20, S22, S30, S32, S35	High	2 studies with minor and 1 study with moderate concerns regarding methodological limitations. 3 studies with minor and 2 studies with moderate concerns regarding coherence. 5 studies with minor and 1 study with moderate concerns regarding adequacy. 4 studies with minor concerns regarding relevance.
End-users’ fascination and curiosity about the robot’s design and features contributed to a positive atmosphere and attitude.	S1, S20, S30	Moderate	1 study with moderate concerns regarding methodological limitations and coherence. 2 studies with minor concerns regarding adequacy. 1 study with minor concerns regarding relevance.
**F4: INTUITIVENESS**	**8 studies**		
End-users were appreciative of robots that were user-friendly and simple.	S1, S5, S6, S8, S11, S12, S14, S32	High	2 studies with minor and 1 study with moderate concerns regarding methodological limitations. 2 studies with minor and 2 studies with moderate concerns regarding coherence. 4 studies with minor and 2 with moderate concerns regarding adequacy. 6 studies with moderate relevance.
End-users were more inclined to operate and integrate the robot without experiencing difficulties.	S1, S5, S8	Moderate	1 study with minor concerns regarding methodological limitations and coherence. 2 studies with minor concerns regarding adequacy. 1 study with minor concerns regarding relevance.
User-friendliness and simplicity are related to certain design elements and features, such as touch screens, menu design, overall interface, and operation of the robot by both verbal and non-verbal communication.	S1, S6	Low	1 study with moderate concerns regarding methodological limitations, coherence, and adequacy. 1 study with minor concerns regarding relevance.
**F5: ASSISTANCE AND USEFULNESS**	**19 studies**		
The robot’s ability to offer utility in healthcare practices impacted attitude levels and perceptions among end-users.	S1, S2, S3, S5, S7, S8, S9, S14, S15, S17, S19, S22, S25, S27, S28, S29, S33, S38, S39	High	5 studies with minor and 1 study with moderate concerns regarding methodological limitations. 10 studies with minor and 2 with moderate concerns regarding coherence. 9 studies with minor and 3 studies with moderate concerns regarding adequacy. 5 studies with minor and 5 studies with moderate concerns regarding relevance.
End-users were appreciative of the robot’s utility, due to a reduction in workload and enabled more time to provide care.	S2, S3, S19, S22, S25, S28, S33	High	1 study with moderate concerns regarding methodological limitations. 3 studies with minor and 1 study with moderate concerns regarding coherence. 3 studies with minor and 2 studies with moderate concerns regarding adequacy. 2 studies with minor and 2 studies with moderate concerns regarding relevance.
End-users would be willing to cope with inconveniences in practice if they experienced a utilitarian impact.	S15, S17, S29, S38	Moderate	1 study with minor and 1 study with moderate concerns regarding methodological limitations. 3 studies with minor and 1 study with moderate concerns regarding both coherence and adequacy. 1 study with minor and 2 studies with moderate concerns regarding relevancy.
**F6: FAMILIARITY**	**13 studies**		
Experience and usage in practice had a positive impact on the end-user’s perception of the robot’s utilitarian value.	S1, S7, S8, S12, S13, S15, S16, S18, S25, S28, S36, S38	High	3 studies with minor concerns regarding methodological limitations. 4 studies with minor and 3 studies with moderate concerns regarding both coherence and adequacy. 3 studies with minor and 3 studies with moderate concerns regarding relevancy.
Initial attitudes of scepticism and neglect decreased once the end-users had become familiar with the robot.	S8, S12, S13, S15, S18, S25, S28, S34, S38	High	2 studies with minor concerns regarding methodological limitations. 2 studies with minor and 1 study with concerns regarding coherence. 4 studies with minor and 1 study with moderate concerns regarding adequacy. 3 studies with minor and 1 study with moderate concerns regarding relevance.
Experiencing robots’ opportunities and limitations in real time had a decreasing impact on fear of being replaced.	S1, S7	Low	No concerns.
**F7: ENHANCING HEALTHCARE PRACTICES**	**14 Studies**		
End-users were appreciative of the robot’s inferior capabilities and broader impact on healthcare.	S1, S2, S4, S7, S8, S16, S18, S21, S22, S23, S25, S27, S28, S33	High	4 studies with minor concerns regarding methodological limitations. 4 studies with minor and 3 studies with moderate concerns regarding coherence. 5 studies with minor and 2 studies with moderate concerns regarding adequacy. 4 studies with minor and 3 studies with moderate concerns regarding relevance.
End-users were appreciative of the robots’ effectiveness and efficiency, as they improved quality of care.	S1, S2, S4, S8, S16, S18, S22, S25, S27, S28, S33	High	4 studies with minor concerns regarding methodological limitations. 4 studies with minor and 3 studies with moderate concerns regarding coherence. 5 studies with minor and 2 studies with moderate concerns regarding adequacy. 4 studies with minor and 3 studies with moderate concerns regarding relevance.

A total of 14 analytical themes emerged from the analysis and qualitative synthesis of the 40 included studies. Of these 14 themes, seven were identified as barriers and seven as facilitators. **Table 3** visualises the distribution of barriers and facilitators among the 40 included studies. The following two subsections present first the barriers and then the facilitators. The third and fourth subsections structure the barriers and facilitators and analyse the anthropomorphisation of robots in hospital environments.

### 3.1. Barriers

We identified seven of the analytical themes as barriers: (B1) user perceptions; (B2) insufficient knowledge, awareness, and support; (B3) inadequate impact on healthcare practices; (B4) limitations to functionalities and design; (B5) workplace dynamics; (B6) workflow implementation; and (B7) loss of control and authority.

**Table 3:** Mapping of barriers and facilitators per included study. For each of the included studies S1-40, the barriers B1-7 and the facilitators F1-7 are marked by red and green check marks, respectively.

	B1	B2	B3	B4	B5	B6	B7	F1	F2	F3	F4	F5	F6	F7
**S1**	✓	✓	✓	✓			✓			✓	✓	✓	✓	✓
**S2**				✓								✓		✓
**S3**												✓		
**S4**														✓
**S5**			✓								✓	✓		
**S6**		✓									✓			
**S7**	✓	✓			✓			✓	✓			✓	✓	✓
**S8**		✓									✓	✓	✓	✓
**S9**	✓	✓	✓	✓		✓		✓	✓	✓		✓		
**S10**	✓		✓	✓		✓								
**S11**			✓							✓	✓			
**S12**		✓						✓			✓		✓	
**S13**						✓		✓	✓	✓			✓	
**S14**		✓									✓	✓		
**S15**	✓							✓	✓	✓		✓	✓	
**S16**													✓	✓
**S17**					✓							✓		
**S18**													✓	✓
**S19**												✓		
**S20**				✓				✓	✓	✓				
**S21**	✓	✓	✓		✓	✓								✓
**S22**	✓		✓	✓						✓		✓		✓
**S23**		✓	✓		✓	✓	✓		✓					✓
**S24**														
**S25**												✓	✓	✓
**S26**		✓												
**S27**								✓				✓		✓
**S28**		✓	✓	✓		✓						✓	✓	✓
**S29**	✓							✓				✓		
**S30**				✓		✓	✓		✓	✓				
**S31**		✓						✓						
**S32**	✓	✓		✓			✓	✓	✓		✓			
**S33**	✓								✓			✓		✓
**S34**		✓			✓			✓	✓	✓			✓	
**S35**	✓									✓				
**S36**													✓	
**S37**														
**S38**			✓		✓							✓	✓	
**S39**												✓		
**S40**				✓				✓						

#### B1: User perceptions

Out of the 40 included studies, 11 addressed that the utilisation and acceptance of the robots were limited by the hospital employees’ animosity and scepticism towards robots [11,12,43–51]. This scepticism was prevalent in all groups of hospital employees, including nurses, physicians, physical therapists, and pharmaceutical and administrative staff. A major component that contributed to those attitudes was the fear of being replaced, as hospital employees across these groups were worried about their physical labour being delegated to and taken over by robots and consequently losing their jobs [12,43,45,46,49]. Similarly, one study highlighted that healthcare workers were impacted by an underlying fear, even when they recognised the robots’ utility and usefulness [46].

Although fear of being replaced was the predominant argument, two studies further mentioned a decrease in the hospital nurses’ competences as a concern: “If robots help us to do the clinical tasks, we’ll reduce nurses’ clinical skills (…)” [49] and “(…) robots would take over nurses’ jobs and that using robots would reduce nurse skills” [43]. Furthermore, three studies addressed the practical implications associated with these attitudes, i.e., when nurses, physical therapists, and administrative staff reject any usage of robots [12,45,51].

#### B2: Insufficient knowledge, awareness, and support

Concerns regarding the sufficiency of hospital employees’ knowledge, awareness, and support were addressed by 14 out of the 40 studies [1,12,43–45,52–60]. The concern regarding insufficient knowledge was primarily illustrated through limited learning opportunities as well as a poor understanding of the robots’ functions, features, and utility [12,43,44,52,54,57,58]. A number of studies indicated that the lack of knowledge contributed to nurses, physical therapists, and pharmaceutical staff feeling nervous, underprepared, and doubtful of the robots’ utility [12,44,52].

Lack of awareness was identified as another concern, as some hospital employees from all groups barely acknowledged robots’ existence and subsequent utility, due to the lack of initiative and proper introduction [52,56,57,59]. One study highlighted that the lack of awareness led to unfamiliarity and inattentiveness, whereas some hospital employees only gained awareness by observing colleagues’ interactions with robots: “I didn’t pay much attention to the system. I didn’t have time to explore (…) I started using it only after seeing another nurse use it (…) it was then I realised how convenient and useful it is" [56].

A multitude of studies further argued for the necessity of providing extensive support to nurses, physicians, physical therapists, and service staff such that they gain an understanding of the robots they are supposed to work with [12,53,60]. Lack of scheduled time to gain experience and practice with robots was the most frequently mentioned concern, which subsequently was found to lead to both integration failure and a decrease in quality of care [12,53]. The studies further addressed the influence of both hesitant management and subordinates, which had a negative impact towards integrating the robots. Additionally, older hospital employees required more support as they perceived robots as more challenging to interact with compared to their younger colleagues [12,60].

#### B3: Inadequate impact on healthcare practices

A perception that the robots failed to have the expected impact was addressed in 10 out of the 40 studies, which raised concerns and dissatisfaction among hospital employees [1,12,45,47,49,52,59,61–63]. Several studies pointed out that the hospital employees as users did not recognise any noticeable improvements, as they perceived the utility of the robots to be limited [1,45,61].

A multitude of studies highlighted that the implementation of robots led to an increase in workload and the frequency of errors [47,49,52,62,63]. Similarly, a perception of the robots being unreliable influenced how and to what degree hospital employees used them, as they were uncomfortable assigning tasks to the robots due to prior experiences of inconsistencies [59,62]. One particular study highlighted that the robots’ unreliability led to the employees performing the tasks themselves and subsequently refusing any further use of them: “Errors occur, and the robots do not as they are told, so we can’t rely on them to accomplish their missions. If I want something done, I will do it myself” [59].

#### B4: Limitations to functionalities and design

Out of the 40 studies, 10 addressed implications associated with the robots’ inadequate functionalities and design features [1,12,44,45,47,49,50,64–66]. Several studies highlighted how malfunctions and outages led to frustrations among hospital employees, as the robots interfered with task execution and even aggravated issues [12,45,50].

The adaptation to hospitals’ complex facilities and infrastructure was another challenge that hindered the integration and utilisation of robots [1,12,50,66]. One study found that the less-than-optimal integration into the hospital infrastructure led to annoyance and disruptions of the workflow, with one nurse relating her frustrations: “It overrides the elevator because the robot was using it, and I was let out into the basement of the hospital with a patient from recovery and had to get out of there. So, we stood there, and had to wait until the robot had finished” [66].

Limitations to certain design features were further highlighted by several studies, such as ineffective interaction through touchscreens, lack of interactivity, elevated noise levels, excessive heat generation, too small or too bulky size, too short and direct phrases, ineffective interaction through touchscreens and verbal interfaces, and protruding attachments [12,45,47,49,64,65]. Moreover, a few studies argued that high volumes of noise and voice emerging from robots impacted privacy levels. Instances of the latter were particularly significant, as robots with verbal communicative features could lead to problems of confidentiality and privacy violations in the perception of healthcare professionals [44,49,64].

#### B5: Workplace dynamics

Robots’ impact on workplace dynamics has been addressed by six out of 40 studies, which found that robots complicated the distribution of responsibilities and roles when integrated into the hospital employees’ working environment [43,52,54,59,62,67]. Two studies illustrated several conflicting perspectives on the distribution of responsibility in instances of mistakes by robots. The perspectives varied from proposing that hospital employees such as nurses and physicians take responsibility, whereas other perspectives instead suggested that the robotic companies should be responsible and take accountability for errors [43,54].

A few studies indicated that the introduction of robots led to modifications in the workplace dynamics perceived as dissatisfactory, as hospital employees filling various professional roles involuntarily were assigned to new roles and tasks [52,59,62]. This modification led to a hierarchy perceived as unpleasant as particularly nurses and service staff experienced being surveilled and expected to take on new less-favoured tasks [52,59,62]. One study highlighted how this hierarchy influenced some pharmaceutical staff members’ morale and perceived contribution: “Assistants became acutely aware that their primary work of stocking medicines was less valued than the work of the others. As they saw it, even the robot had downgraded their work” [52].

#### B6: Workflow implementation

Out of the 40 studies, seven addressed robots as an obstacle when implemented into routine- and workflow-related practices [1,12,47,52,59,66,68]. Several studies argued that the implementation of robots into workflows decreased the quality of care and disrupted the employees’ daily routines, which led to hospital employees feeling dissatisfied [12,59,66,68]. One study illustrated how nurses and physicians perceived the robots’ utility to be limited within their workflows, as their usefulness was only applicable in selective niche work processes and environments [1].

#### B7: Loss of control and authority

The hospital employee’s concerns about dependency, lack of involvement, and associated perceptions of unreliability were addressed by four out of the 40 included studies [44,45,52,66]. One study argued that the users among the pharmaceutical staff were frustrated with their lack of influence regarding the integration of robots and felt they were losing autonomy as routines were constantly rearranged to accommodate the robots’ limitations [52]. Furthermore, another study addressed that robots would be granted authority by subordinates and managers in the sense that they were given the ability to override hospital employees such as nurses in certain work processes [66].

### 3.2. Facilitators

Seven analytical themes emerged as facilitators: (F1) training and engagement; (F2) transparency; (F3) behavioural and cultural factors; (F4) intuitiveness; (F5) assistance and usefulness; (F6) familiarity; and (F7) enhancing healthcare practices.

#### F1: Training and engagement

Out of 40 included studies, 12 addressed the positive impact of extensive training and engagement on the robots’ perceived utility and overall satisfaction levels among hospital employees [11,12,43,44,46,50,54,55,60,65,68,69]. Multiple studies highlighted how hospital employees benefited from introductory and educational programs. The employees developed a greater understanding of the robots’ features and complexity, often by selecting certain employees as ‘key users’ or specialist supervisors, to ease the process for the remaining employees [12,44,46,50,54,60]. Several studies argued that hospitals must provide an adequate amount of support and resources throughout the training and educational procedures, complemented by freeing employees to develop and participate in public campaigns, consultations, implementation strategies, and active user involvement activities aimed at influencing the perception, intention, and usefulness of the robots [11,12,44,46,50,68,69].

#### F2: Transparency

A total of 10 out of 40 studies highlighted the importance of transparency regarding the robots’ utility, roles and responsibilities, and expectation management [11,12,43,44,48,50,52,54,66,68]. Several of the studies illustrated that the hospital employees appreciated clarity regarding the robots’ limitations and responsibilities, as well as a realistic insight into their potential impact on work processes and roles [11,12,43,44,48,50,54,68]. Three studies emphasised the importance of presenting robots as tools to assist hospital employees in avoiding doubt and uncertainty regarding replaceability, which contributed to increased acceptance among healthcare workers and cleaning staff [12,54,66].

#### F3: Behavioural and cultural factors

Hospital employees’ attitudes, perceptions, and values were addressed by 10 out of the 40 studies and were shown to have an enabling influence on the application and integration of robots within a hospital environment [11,12,44,45,49–51,63,66,68]. These behavioural and cultural factors were illustrated through the elements of motivation, enthusiasm, and a generally positive attitude towards technology, which facilitated the hospital employees to accept and engage with robots [12,44,49–51,68].

The impact of a positive attitude towards technology was especially highlighted in one study, where the nurses’ reflections indicated that robots were likely to be essential within future healthcare practices: “(…) constant learning and lifelong learning in improvements of technology is essential to respond to the alterations in pediatric nursing practice (…) nurses have to maintain current knowledge of highly skilled responses to meet the demands of complex health problems” [49].

Two studies illustrated that nurses and surgeons experienced an alignment between their personal values and norms with the acceptance and positive perceptions towards robots [51,68]. Other facilitating elements were the impact of the hospital employees’ fascination and curiosity regarding the robots’ designs and features, which contributed to a positive atmosphere and attitudes towards the robot [45,50,66].

#### F4: Intuitiveness

Out of the 40 studies, eight argued that robots’ intuitiveness and subsequent ease of use had a positive impact, as the robots would be easier to operate which had a facilitating influence on the integration process [44,45,53,56,57,60,61,63]. Several studies highlighted how user-friendliness and simplicity positively impacted the integration process, as the hospital employees could operate and implement the robots with less effort [45,56,61]. A few studies addressed intuitiveness in relation to certain elements of the robots’ designs and features such as touch screens, menu design, overall interface, and the ability to operate the robot through both verbal and non-verbal communication [45,53].

#### F5: Assistance and usefulness

Out of the 40 included studies, 19 highlighted how robots’ usefulness and ability to assist hospital employees in healthcare practices had a positive impact on attitude levels and perceived utility [1,11,12,43,45,46,48,49,56,57,61,62,64,67,69–73]. Multiple studies addressed the hospital employees’ appreciation for the robots’ utility, as it facilitated a reduction in their workload, enabling the employees to spend more time providing care [1,48,49,64,70–72].

One study, in particular, highlighted the robots’ usefulness in healthcare practices involving children, which nurses greatly appreciated due to the difficulties they often experienced with such patients: “The participants felt that care robots could enhance these patients’ cooperation by being friendly and engaging with them (…) All too often, it’s hard to get children to play along. So, I think [robots] could persuade children to cooperate while we do our jobs” [72]. Furthermore, several studies addressed the significance of robots being useful in healthcare practices as being the single most important component. Accordingly, the employees would be able to cope with the robots’ inconveniences in practice, if they instead experienced a utilitarian impact [11,46,62,67].

#### F6: Familiarity

Out of the 40 studies, 13 addressed the importance of familiarity built on experience and usage in practice as having a facilitating impact on the hospital employees’ perception of the robots’ utility [1,11,12,43,45,56,60,62,68,70,74–76]. Several studies addressed attitudes of both scepticism and neglect among hospital employees toward their relationships with robots. However, once the employees had become familiar with the robots, through engagement in practice and experiencing their utility, they acknowledged an increase in acceptance and satisfaction levels [1,11,56,60,62,68,74,75,77]. Similarly, two studies highlighted that knowledge, reliability, and satisfaction levels increased over a longer period, which indicates that both hospitals and employees such as nurses and service staff must be patient for robots to reach their full utilitarian potential [60,62]. Becoming familiar with the robots further ameliorated the fear of being replaced among some healthcare workers and administrative staff as they experienced the robots’ opportunities and limitations in real time, which contributed to a less unpleasant experience when using and cooperating with the robots [43,45]. One study also acknowledged the significance of integrating robots as early as possible into workflows and standardising subsequent workflow processes to avoid uncertainty among end-users such as nurses and physical therapists, as well as risks towards safety measures [12].

#### F7: Enhancing healthcare practices

Out of the 40 studies, 14 addressed how the implementation of robots had a positive impact on hospital employees, as they appreciated the robots’ capabilities and broader impact on hospitals [1,9,43,45,48,49,52,56,59,64,69,70,74,76]. Several studies highlighted that hospital employees were satisfied with the robots’ efficacy and efficiency, as they perceived them to provide a significant improvement in the quality of care [1,9,45,48,49,56,64,69,74,76,77]. Several studies acknowledged that robots could execute healthcare practices in an improved manner simply not possible for hospital employees due to restrictions in time and capabilities [9,45,49,56,59,64].

Multiple studies also indicated that the implementation of robots influenced unforeseen areas of the execution of healthcare practices, with a positive impact on overall satisfaction levels among hospital employees. These studies addressed the following four areas: bringing neutrality to challenge hierarchical culture and power dynamics, improving hospital employees’ well-being and reducing turnover intention, increasing legitimacy and professionalisation within the healthcare domain, and resolving long-term workforce problems by reducing workload for low-skilled jobs [1,9,52,74].

#### 3.3. Structuring barriers and facilitators

In order to structure the total of 14 themes distributed to seven barriers and seven facilitators we identified, we qualitatively coded the themes according to whether they engage with the *individual*, the *organisational*, the *technological* sphere, or any combination thereof. Structuring medical and technical innovation through a focus on these three (and sometimes further) spheres has a long history in innovation management [78] and medical sociological research [79]. In the case of the integration of robotic technology in hospital environments, the individual sphere encompasses the hospital employees as the users, the organisational sphere is comprised of the hospital and its administrative and medical procedures as an institution, and the technological sphere is constituted by the robotic technologies and the other technologies that integrate with it.

**Fig 4:**
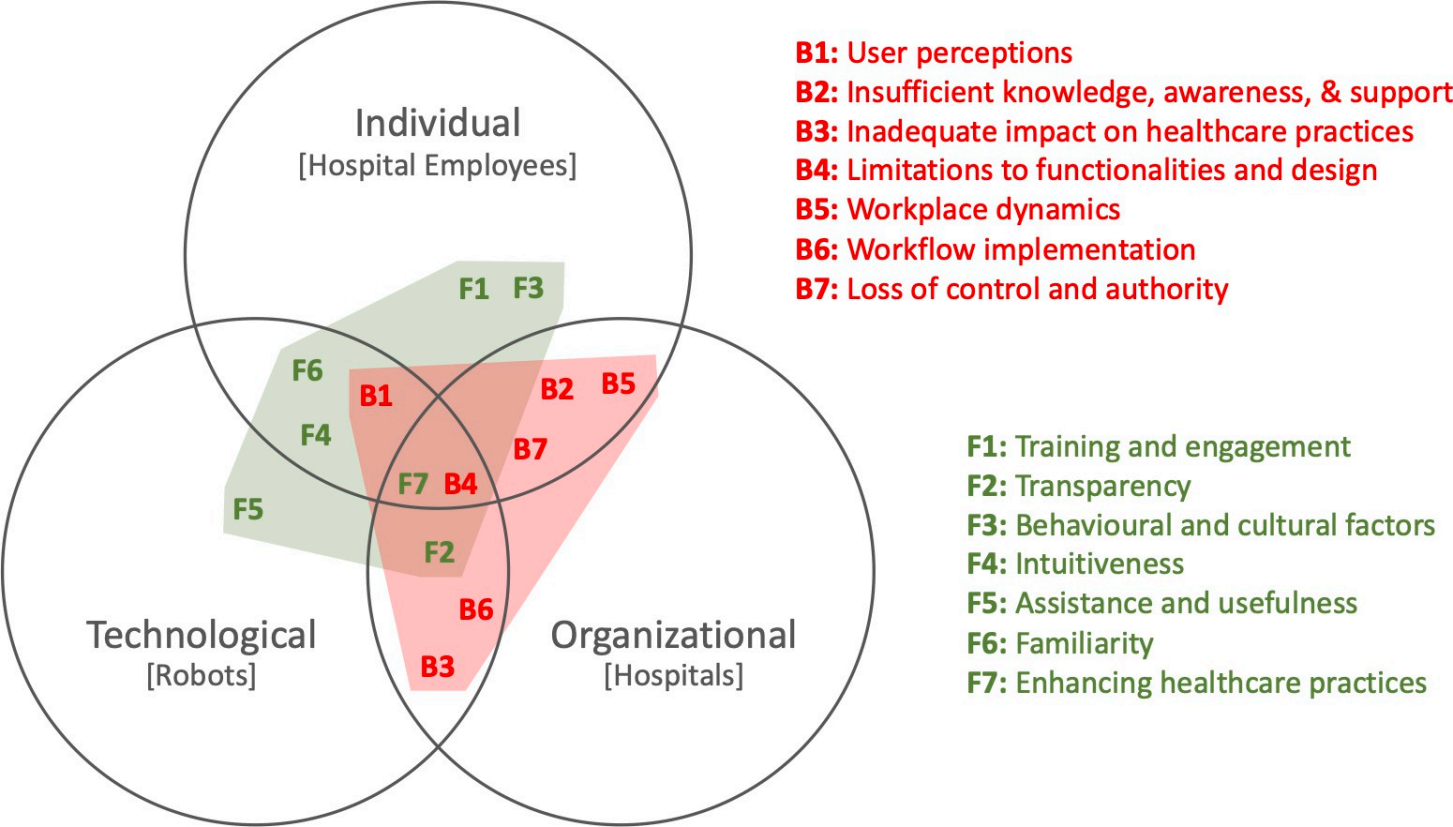
Barriers and facilitators in the individual, organisational, and technological spheres.

**Fig 4** employs a 3-set Venn diagram to illustrate the engagement of the barriers **B1** to **B7** and facilitators **F1** to **F7** with the three spheres. Facilitator **F6**, for example, is placed in the intersection of the individual and technological sphere while barrier **B6** is placed in the intersection of the technological and organisational sphere.

As a first observation, consider that barriers arise in the intersections of two or more of the spheres (7 out of 7) while some facilitators also engage with only a single sphere (3 out of 7). Furthermore, the facilitators intersecting more than one sphere are mostly located between the individual and the technological sphere (3 out of 4) while the intersecting barriers nearly all involve the organisational sphere (6 out of 7).

In order to visualise this difference in engagement between barriers and facilitators, we added the convex hulls of the barriers (red polygon) and the facilitators (green polygon) to **Fig 4**. This visualisation crystallises one of the main observations emanating from our analysis–that there is a misalignment between the barriers and facilitators of robotic technologies in hospital environments. The main takeaway message from this observation is the need for future research to dive deeper into how the barriers engaging with the organisational sphere can be effectively addressed, particularly in the intersection with the individual sphere. In other words, research aimed at understanding how to address the barriers of robots in hospitals should focus on the implications of the integration of this technology on the interplay between hospital employees and the procedures and workflows of the hospital rather than narrowly on the capabilities and limits of the technology.

### 3.4. Anthropomorphisation

Anthropomorphisation has been a frequent subject within the examination of human-robot interaction. Unsurprisingly, the subject occurred several times throughout the included studies, in relation to the hospital employees’ perspectives, arguments, and behaviours. The terminology of ‘anthropomorphisation’ references the tendency to incorporate elements of human qualities within an object, such as either a machine or a robot [66]. A total of five out of the 40 studies addressed anthropomorphisation in relation to the hospital employees’ integration and utilisation of robots [45,59,66,69,80].

Two studies addressed concerns over the impact of ‘anthropomorphisation’, as human-like traits led to the nurses, physicians, and cleaning staff perceiving the robots as either uncomfortable, less accessible, or too agentic [66,81]. These concerns were illustrated through two perspectives: patterns of mistrust and unease towards robots being presented as something they are not (e.g., a human or animal), and robots being imbued with too high levels of authority and agency within certain work processes. Four studies addressed the favourable impact of anthropomorphisation, particularly through the incorporation of domestication-related activities to integrate robots into the hospital employees’ community [45,59,66,80]. The studies illustrated that levels of morale and appreciation increased among hospital employees when domestication-related activities were incorporated into the usage of robots. Examples of such are greeting the robot when leaving work, giving the robot a name, the robot’s verbal communication features accommodating local dialects, and interacting with the robot to make it feel part of the companionship among health and service workers [59,66,80].

The findings’ lack of volume and coherence on this matter indicates that it is complicated to determine whether anthropomorphisation constitutes a barrier or a facilitator. Thus, hospitals and robotic companies must be cautious of both the lack of cohesiveness among the findings, as well as the overall minor presence of anthropomorphisation within the eligible studies, if these findings are to be applied in practice. These arguments further indicate that the research subject of ‘anthropomorphisation’ in relation to hospital employees as end-users is underexplored. Therefore, scholars should elaborate on the subject in future studies to develop a more comprehensive understanding.

## 4. Discussion

In the following, we first consider the trustworthiness of robotic systems in hospital environments. We then discuss privacy and confidentiality, before reflecting on the limitations and implications of our review.

### 4.1. Trust and trustworthiness of robotic systems

Trust in an agent (a robotic system in this case) has been described as a property of the human user, and conversely, trustworthiness as a property of the robotic agent [82]. A recent narrative review [83] highlighted the factors influencing the development of trust in robotic agents, considering three over-arching categories: *human factors*, *robot factors*, and *contextual factors*.

Our findings affirm that *human factors* play key roles in the integration of robots in hospital environments. These factors rely on four main characteristics: sociodemographic traits, attitudes towards robots, acceptance/preference of robots, and prior experience with technology and/or robotic agents [83]. A positive attitude towards technologies, in general, and robots, in particular, (facilitator F3 on behavioural and cultural factors) increases acceptance/preference of robots [12,44,49–51,68]. Conversely, the fear of being replaced (barrier B1 on user perceptions) hinders acceptance, leading hospital employees such as nurses, physical therapists, and administrative staff to reject robots [12,45,51]. A lack of experience with robotic technologies (barrier B2 on insufficient knowledge, awareness, and support) further undermines human trust in robots [12,44,52] unless addressed by training (facilitator F1 on training and engagement) of hospital employees [11,12,43,44,46,50,54,55,60,65,68,69].

Beyond the attitudes of hospital employees and management aspects such as the allocation of resources for training, our findings also underscore the importance of the utility of the robotic technologies. These *robot factors* are related to the design, construction, and performance of the robot [83]. Issues with the design and construction of robots [12,45,47,49,64,65] and doubts about their suitability for complex hospital environments [1,12,50,66] undermine their trustworthiness (barrier B4 on limitations to functionalities and design). While perceptions of positive impact on healthcare processes (facilitator F5 on assistance and usefulness) may offset such design flaws [11,46,62,67], when end-users experience that robots make errors and increase rather than decrease workload, this undermines the robots’ trustworthiness through doubts about their performance [1,12,45,47,49,52,59,61–63].

Hospital employees also represent a group of professional end-users, which adds further aspects to their development of trust in robotic technologies. For patients as the other significant group of end-users, trustworthiness is primarily grounded in being able to trust that robots operate fairly, safely, and reliably while providing accurate care [84]. Hospital employees as end-users aspire to deliver the best service possible to hospitalized patients, requiring high levels of confidence in the tools and collaborative partners they work with. Thus, for hospital employees, transparency and explainability are paramount when deploying robots to ensure consistent and trustworthy results [85] as they allow the users to judge whether an automatic decision makes sense from a human perspective instead of blindly trusting an autonomous system.

Trustworthiness of robots in hospital environments, thus, also depends on *contextual factors* such as teamwork and in-group membership, communication, and shared mental models [83]. When the distribution of responsibility in a team of humans and robots is perceived as unclear or dissatisfactory [43,52,54,59,62,67], this complicates teamwork (barrier B5 on workplace dynamics). Our findings indicate that successful in-group membership of and communication with robots builds on transparency (facilitator F2) regarding their limitations, responsibilities, and impact [11,12,43,44,48,50,54,68], particularly when the functionalities and decision-making of the robots are perceived as understandable and manageable by the end-users. These contextual factors for trustworthiness are further reinforced by the intuitiveness (facilitator F4) of interacting with the robots [44,45,53,56,57,60,61,63].

### 4.2. Privacy and Confidentiality

Hospital employees as end-users must also feel confident that data are collected, stored, processed, and managed privately and securely. Robots placed in healthcare inherently work with sensitive data. Particularly autonomous robots require a series of sensors to operate and as such collect a series of data points, many of which are considered sensitive including, but not limited to, audio and video data. A selection of seven of the included studies briefly touches upon privacy and security concerns, typically from an ethical rather than practical perspective [9,43,44,46,49,50,53].

From an ethical perspective, data collection raises several questions such as: how informed consent is gathered and whether it is needed in the first place, how patients and hospital employees feel about being under surveillance, and with whom the data can be shared [46,53]. Nevertheless, the practical opportunities provided by the gathered data are significant and can potentially improve the robots’ presence and efficacy.

AI plays a major role in creating autonomous robots, but privacy challenges arise when the data collected by in-field robots is used to improve existing AI models. In a traditional AI training setting, the data would be collected from all the robots to a central server where the model is trained. However, the transfer of data is one of the areas where privacy violations can be incurred. To accommodate these issues, federated learning has been proposed to allow AI model training without the sensitive data ever leaving the edge device, in this case, the robot [86,87]. In a federated learning setting, the AI model training is distributed to several robots and managed by a central server. Each robot trains a model locally and only transmits the learned model to the central server. All the local models are aggregated into a single global model that is distributed to all edge devices.

Some robots have communicative capabilities that allow for the transmission of information from the robot to a human. In a hospital setting, this communication, whether it is auditory or visual, increases the risk of disclosing confidential information about patients [44,49,64]. Unauthorised individuals may overhear or read the disclosed information against the patients’ or hospital employees’ wishes.

Both issues with the transmission of information and the collection of sensitive data constitute barriers that have not been explored to a satisfactory level by the included studies. Some issues can be mitigated by taking appropriate actions such as using privacy-enhancing techniques, while others require careful design considerations. Nevertheless, as robot technology advances, privacy challenges should be approached from the perspective of Privacy by Design, which aims to integrate privacy as a fundamental property of the design and lifecycle of technologies [88].

### 4.3. Limitations and implications

While the findings of this study reveal a notable divergence in hospital employees’ perceptions, attitudes, and behaviours towards the use of robots, the analysis of the included studies did not allow for comprehensively distinguishing and comparing the differences between the user perceptions of different groups of hospital employees due to an uneven coverage of these groups. A few studies indicated that while negative user perceptions may affect one group of employees, positive user perceptions may influence another group. Future research is required to build an in-depth understanding of how the barriers and facilitators we identified relate to different groups. Similarly, our findings do not necessarily contextualise to the unique hospital environments and specific types of robots. More research is needed such that the barriers and facilitators we identified can be refined to take into account the distinct features, goals, and intended users associated with each type of robot. Moreover, another limitation was a bias towards high-income countries that carried from the initial articles screened through to the included sample, which may be assumed to limit the generalizability to settings in low-income countries. We underline the necessity of future research in this direction, both regarding the integration of robots in hospitals in low-income countries and how economic and sociocultural disparities impact this integration. A final limitation of this scoping review is that only one database was searched. We cannot fully exclude that other databases might contain further relevant articles.

The findings we presented have implications for the feasibility of integrating robots in hospital environments. Robots are typically meant to minimise resource usage and save costs in hospital environments. Our analysis reveals, though, that the initial integration period may demand substantial resources beyond the initial cost of acquiring the robotic systems. Hospitals need to be prepared to allocate resources upfront in order to maximise the long-term benefits. Our analysis also indicates the importance of bridging the gap between robotic companies and hospitals by involving hospital employees in all development stages of new robotic technologies. Allowing hospital employees to voice their expectations and concerns and to contribute with insights to the design has the potential to improve the alignment of robots with the target hospital environments.

## 5. Conclusion

Through a scoping literature review, we reviewed 40 pertinent user-centric studies derived from an original set of 501 screened original research articles. We identified, analysed, and structured 14 themes into seven barriers and seven facilitators of the integration of robotic technologies in hospital environments. We revealed a notable misalignment between these barriers and facilitators regarding their engagement with individual, organisational, and technological aspects, with organisational factors being at the core of most barriers.

Consequently, we suggest that future research on understanding and addressing these barriers explores the interplay between hospital employees as professional users and institutional procedures and workflows, as well as the ambivalent role of the anthropomorphisation of hospital robots and emerging concerns related to privacy and confidentiality issues raised by communicative robots.

This scoping review provides a perspective that highlights the importance of transcending debates solely focusing on the capabilities and limitations of new healthcare technologies, instead advocating to shed light on the intricate process of integrating new technologies into complex hospital environments. This perspective contributes valuable insights for the future of healthcare innovation, with a focus on addressing the complexities arising at the nexus of individual, organisational, and technological aspects of healthcare delivery.

## Supporting information

S1 Appendix PRISMA checklist. (PDF)

S1 Table Search terms and their Boolean operators. (DOCX)
