# Supplementary material for: New colleague or gimmick hurdle? A user-centric scoping review of the barriers and facilitators of robots in hospitals
Source: PLOS Digit Health. 2024 Nov 11;3(11):e0000660. doi: 10.1371/journal.pdig.0000660 (PMC11554139; doi:10.1371/journal.pdig.0000660)
Supplement: S1 Table — (DOCX) [file pdig.0000660.s002.docx]

**S1 Table.** Search terms and their Boolean operators.

| **Boolean operator** | **Search terms** (* denotes truncation, matches any ending) |
| --- | --- |
| **OR** (disjunctive, match at least one) | attitude*, perspective*, point of view, nurse*, healthcare worker, healthcare-professional, health worker, user experience, barrier, challenge, user perspectives, user*, facilitators, implementation, enabler*, healthcare, influenc*, key factors, impact*, benefits, problems, perception, adapt*, compliance, behavior*, accept*, assistive technology, opinion* |
| **AND** (conjunctive, match all) | robots, hospital |
